# Selected commensals educate the intestinal vascular and immune system for immunocompetence

**DOI:** 10.1186/s40168-022-01353-5

**Published:** 2022-09-28

**Authors:** Rossana Romero, Agnieszka Zarzycka, Mathieu Preussner, Florence Fischer, Torsten Hain, Jan-Paul Herrmann, Katrin Roth, Corinna U. Keber, Kushal Suryamohan, Hartmann Raifer, Maik Luu, Hanna Leister, Wilhelm Bertrams, Matthias Klein, Hosam Shams-Eldin, Ralf Jacob, Hans-Joachim Mollenkopf, Krishnaraj Rajalingam, Alexander Visekruna, Ulrich Steinhoff

**Affiliations:** 1 Institute for Medical Microbiology and Hygiene, Philipps-University Marburg, Marburg, Germany; 2 Present Address: Cell Biology Unit, University Medical Center, Johannes Gutenberg University, Mainz, Germany; 3 Pfizer GmbH, Berlin, Germany; 4 Department of Environmental Immunology, Helmholtz Centre for Environmental Research — UFZ, Leipzig, Germany; 5 Institute of Medical Microbiology, Justus Liebig University Giessen, Giessen, Germany; 6 Partner Site Giessen-Marburg-Langen, German Center for Infection Research (DZIF), Justus Liebig University Giessen, Giessen, Germany; 7 Center for Tumor Biology and Immunology, Philipps University Marburg, Marburg, Germany; 8 Pathology, University Hospital of Giessen and Marburg (UKGM), Marburg, Germany; 9 Medgenome, Inc., San Mateo, CA USA; 10 Flow Cytometry Core Facility, Philipps University Marburg, Marburg, Germany; 11 Medizinische Klinik und Poliklinik II, Universitätsklinikum Würzburg, Würzburg, Germany; 12 Institute for Lung Research, Universities of Giessen and Marburg Lung Center (UGMLC), Philipps University Marburg, Marburg, Germany; 13 Institute for Immunology, University Medical Center Johannes Gutenberg University, Mainz, Germany; 14 Tierexperimentelle Einrichtung, Philipps University of Marburg, Marburg, Germany; 15 Department of Cell Biology and Cell Pathology, Philipps University of Marburg, Marburg, Germany; 16 Department of Immunology, Max Planck Institute for Infection Biology, Berlin, Germany; 17 Biomedical Research Center (BMFZ), Institute for Medical Microbiology and Hygiene, University of Marburg, Hans Meerwein Straße 2, 35043 Marburg, Germany

**Keywords:** Microbial consortia, Commensal imprinting, Asymptomatic infection, Endothelial cells, Blood vessel development, Intestinal maturation, Genome-guided microbiota, Neutrophils, *C. rodentium*, Oligo-Mouse-Microbiota, Colonization resistance, Enteric pathogen

## Abstract

**Background:**

The intestinal microbiota fundamentally guides the development of a normal intestinal physiology, the education, and functioning of the mucosal immune system. The *Citrobacter rodentium*-carrier model in germ-free (GF) mice is suitable to study the influence of selected microbes on an otherwise blunted immune response in the absence of intestinal commensals.

**Results:**

Here, we describe that colonization of adult carrier mice with 14 selected commensal microbes (OMM^12^ + MC^2^) was sufficient to reestablish the host immune response to enteric pathogens; this conversion was facilitated by maturation and activation of the intestinal blood vessel system and the step- and timewise stimulation of innate and adaptive immunity. While the immature colon of *C. rodentium*-infected GF mice did not allow sufficient extravasation of neutrophils into the gut lumen, colonization with OMM^12^ + MC^2^ commensals initiated the expansion and activation of the visceral vascular system enabling granulocyte transmigration into the gut lumen for effective pathogen elimination.

**Conclusions:**

Consortium modeling revealed that the addition of two facultative anaerobes to the OMM^12^ community was essential to further progress the intestinal development. Moreover, this study demonstrates the therapeutic value of a defined consortium to promote intestinal maturation and immunity even in adult organisms.

Video Abstract

**Supplementary Information:**

The online version contains supplementary material available at 10.1186/s40168-022-01353-5.

## Introduction

After birth, mammals successively acquire microbes on their mucosal surfaces with the most numerous communities residing in the gut. These slowly developing microbial communities not only help to digest our food and produce important bioactive substances but are essential for the maturation of immune cells and organ structures [1]. While full maturation of the human intestinal microbiota requires several years depending on nutrition, social contacts, and pets [2], microbial colonization between birth and weaning is essential for normal development and immune reactivity [3]. This period is called *window of opportunity*, as the cross talk between commensals and their host during this time has long-term consequences for a healthy or pathological immune reactivity [4]. In contrast to early life, our understanding as to what extent commensal bacteria may affect the morphology and immune function of an adult organism is still incomplete. To this end, GF animals provide an ideal analytical tool, as the lack of commensals has profound and lifelong effects on the structural and functional development of the mucosal immune system [5]. Although transfer of a normal intestinal microbiota has been shown to correct many developmental and immune-related cellular pathways in these mice [6, 7], the complexity and variability of the microbiota make it difficult to assess whether tissue- and immune-related transcriptional responses after conventionalization are similar to normally raised specific-pathogen-free (SPF) mice [8]. Therefore, understanding the impact of defined bacteria or limited microbial consortia on maturation and development of tissue structures and the immune system would be a big advantage for the targeted intervention of acute and persisting infections.

For several pathogens, an unknown fraction of infected individuals is able to spread the disease even though they are free of symptoms [9]. As asymptomatic carriers handicap the control of pathogen transmission and unaware antibiotic treatment favors the emergence of drug-resistant strains, investigation of possible causes for carrier development is of utmost importance [10, 11].

We here analyzed the carrier phenomenon by using GF mice infected with *C. rodentium*, a murine pathogen that belongs to the family of extracellular enteric pathogens related to human enteropathogenic (EPEC) and enterohemorrhagic (EHEC) *Escherichia coli* [12, 13]. While animals with a normal laboratory microbiota (SPF) eradicate the pathogen, *C. rodentium* downregulates its virulence factors [14] and persists lifelong asymptomatically in GF mice which, similar to asymptomatic human carriers, are able to spread the infection.

As the constellation of intestinal microbes that make up an individual’s microbiome is highly unique [15], subtractive manipulation of a complex microbiota is challenging compared to the additive strategy which can also be applied in GF animals. A recently described model microbiota (Oligo-Mouse-Microbiota, OMM^12^) [16] alone or together with rationally selected microbes was used for their potential to convert adult *C. rodentium*-carrier mice into responder animals. We provide evidence that colonization of adult GF mice with OMM^12^ induces maturation of intestinal blood vessels and activation of the immune system to support neutrophil migration to reach the intestinal lumen where *C. rodentium* resides. Enforcing OMM^12^ with two rationally selected microbes (*E. coli* and *Citrobacter amalonaticus*, MC^2^) further enhanced intestinal development and provided GF mice colonization resistance and immunity against *C. rodentium* comparable to conventional mice.

Thus, we hypothesized that the lack of defined intestinal microbes may contribute or favor the development of a pathogen carrier status which might be corrected by rational microbiota-based therapy.

## Results

### Fourteen commensals induce C. rodentium elimination by enhancing colonic neutrophil recruitment

**Fig. 1:**
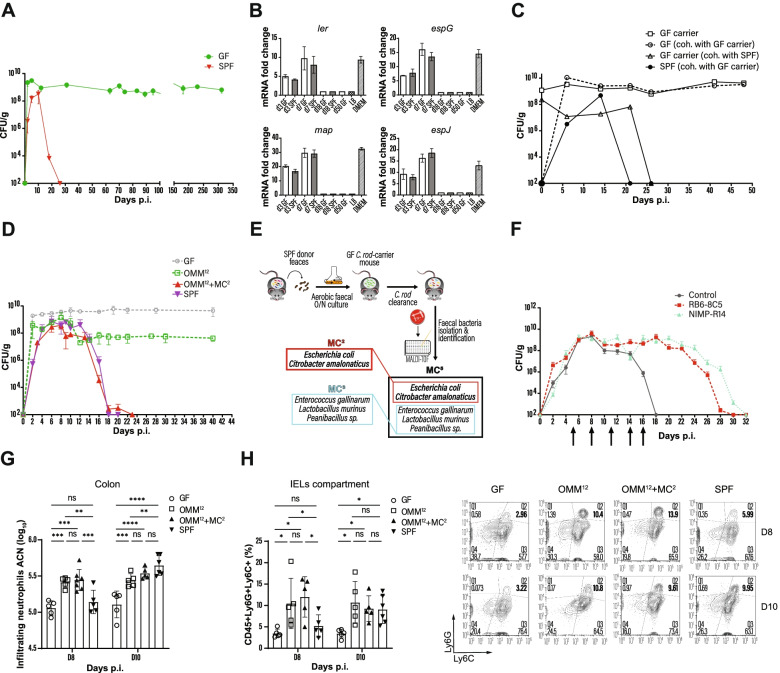
Commensals induce *C. rodentium* elimination by enhancing colonic neutrophil recruitment. **A** Course of *C. rodentium* infection in GF and SPF. **B** Kinetic of virulence gene expression in *C. rodentium*. **C** CFUs of *C. rodentium* in GF or SPF mice cohoused with *C. rodentium*-carrier mice. **D** Fecal CFUs in indicated mice after infection with *C. rodentium*. **E** Schema for identification of facultative anaerobic bacteria from the fecal microbiota of SPF mice. **F** *C. rodentium*-infected SPF mice were treated i.p. with neutrophil-depleting antibodies at indicated time points (arrows), and fecal CFUs were quantified. **G** Absolute cell number (ACN) of infiltrating neutrophils into the colon of mice at days 8 and 10 p.i. **H** Neutrophil frequencies in the colonic intraepithelial compartment at days 8 and 10 p.i. Animals were orally infected with (10^9^ CFU/ml) *C. rodentium*. Data shown are mean ± SD of 2–3 independent experiments; each symbol represents the individual value for one mouse (**D** and **E**). One-way ANOVA was used: ****p < 0.0001; **p ≤ 0.01; *p ≤ 0.05; ns, not significant

Monitoring the infection of GF mice with *C. rodentium* for more than 1 year, we observed that animals devoid of microbiota turned into asymptomatic carriers, despite high bacterial loads (Fig. 1A). As recently described for EHEC and EPEC strains [17], the lack of *C. rodentium* pathogenicity was also associated with a downregulation of virulence genes, from day 18 p.i. (Fig. 1B), yet long-term persisting *C. rodentium* was still able to infect naïve mice (Fig. 1C).

Starting from the premise that conventionalization of GF mice triggers the elimination of *C. rodentium* (Fig. S1A), we searched for a defined bacterial community that confers colonization resistance and immunity in *C. rodentium* carriers by testing the OMM^12^ microbiota model. OMM^12^-colonized mice infected with *C. rodentium* revealed that after the initial rapid multiplication, the pathogen load dropped markedly by day 10 and remained constant until the end of the experiment (Fig. 1D). Although OMM^12^ caused a 200-fold pathogen reduction compared to GF mice, this consortium was not sufficient to mimic a complex microbiota (Fig. 1D). As facultative anaerobic bacteria are underrepresented in the OMM^12^ consortium, we mined the microbiota of SPF mice for strains that together with OMM^12^ facilitate the clearance of *C. rodentium* in carrier mice as outlined in Fig. 1E. To lower the microbial complexity, feces from SPF mice were cultivated under aerobic conditions and transferred into *C. rodentium* carriers. As recipients of these fecal microbiota transplants (FMT) were able to eliminate the pathogen (Fig. S1B), we searched for commensals that were still present in these mice after pathogen elimination. A consortium of five facultative anaerobes was identified (MC^5^: *E. coli*, *C. amalonaticus*, *Enterococcus gallinarum*, *Lactobacillus murinus*, and *Paenibacillus* sp.) (Fig. 1E). Remarkably, enrichment of OMM^12^ with MC^5^ (OMM^12^ + MC^5^) was able to induce pathogen clearance (Fig. S1C). To further reduce the complexity, we selected *E. coli* and *C. amalonaticus* (MC^2^), as both bacteria have been reported to induce colonization resistance against *C. rodentium* [14, 18]. OMM^12^ mice associated with MC^2^ prior to *C. rodentium* infection cleared the pathogen with kinetics similar to SPF and OMM^12^ + MC^5^ mice (Figs. 1D and S1C). It should be noted that clearance of *C. rodentium* in OMM^12^ + MC^2^ mice was specifically promoted by MC^2^ bacteria, as OMM^12^ mice colonized with the remaining 3 facultative anaerobes from MC^5^, *E. gallinarum*, *L. murinus*, and *Paenibacillus* sp. (MC^3^), failed to eliminate the infection, similar to OMM^12^ mice (Fig. S1C).

**Fig. 2:**
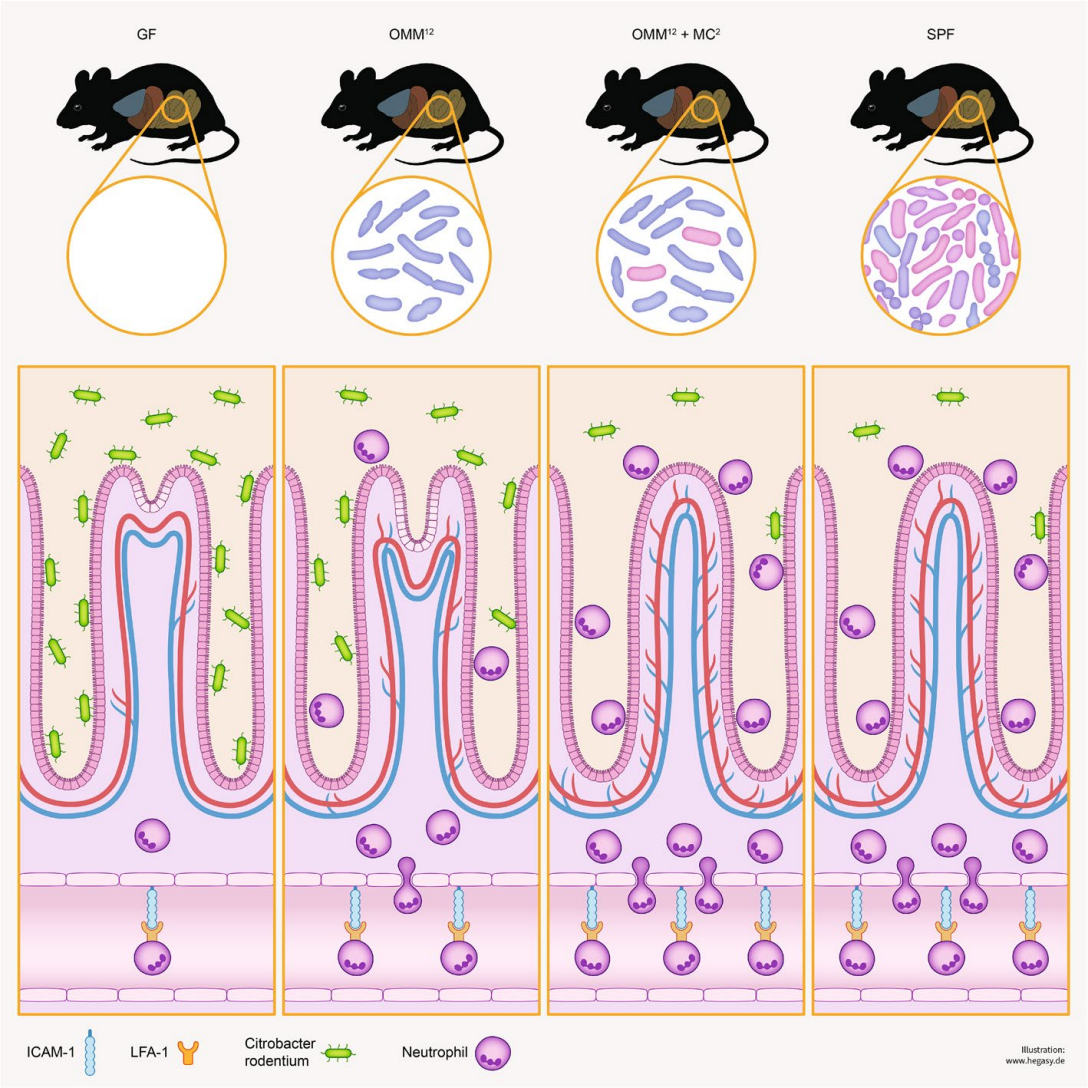
Influence of commensals on maturation of the vascular and immune system. Sketch shows crypts, blood vessels, *C. rodentium* and neutrophils under different colonization conditions. GF mice have an immature colon and when infected with *C. rodentium*, they become lifelong carriers with high pathogen titers and low numbers of neutrophils in the colon. Colonization of GF animals with the OMM^12^ microbiota prior to *C. rodentium* infection leads to partial maturation of the colonic blood vessels and crypts. Furthermore, OMM^12^ colonization activates endothelial cells allowing neutrophils to extravasate into the lamina propria and lumen of the colon, with partial elimination of *C. rodentium*. Addition of MC^2^ to the OMM^12^ consortium further enhances tissue maturation of the colon allowing clearance of *C. rodentium* comparable to SPF mice

Neutrophils are essential for the clearance of *C. rodentium* infection [19]. Accordingly, transient antibody-mediated depletion of neutrophils in SPF mice led to delayed pathogen clearance (Fig. 1F). Here, we examined the impact of the microbiota on neutrophil migration into the colon during the infection. *C. rodentium*-infected GF mice showed low neutrophil numbers in the colon that peaked at day 8 p.i. and declined afterwards (Fig. S1D). SPF mice displayed a similar neutrophil migration pattern until day 8 p.i., followed by a sharp increase at day 10 p.i., a time point that coincides with the decline of *C. rodentium* titers in SPF mice (Fig. S1F and Fig. 1D). Since these findings suggest that migration of neutrophils into the colon is controlled by the intestinal microbiota rather by the pathogen (Fig. 2), neutrophil numbers were examined in OMM^12^ and OMM^12^ + MC^2^ mice. In comparison with GF, OMM^12^ and OMM^12^ + MC^2^ mice showed significantly increased infiltrations of neutrophils on day 8 p.i. which was comparable to SPF mice on day 10 p.i. (Fig. 1G), demonstrating that OMM^12^ colonization was sufficient to induce a robust migration of neutrophils into the colon on both days (Fig. 1G).

*C. rodentium* is an extracellular pathogen that adheres to the luminal site of the intestinal epithelium [20]; thus, neutrophils must migrate through the lamina propria (LP) and cross the epithelial barrier to reach the gut lumen for pathogen engulfment [19]. Therefore, we studied the influence of microbial consortia on neutrophil migration into the colonic LP and those migrating through the epithelial lining during infection. As previously reported for GF and SPF mice [14], the frequencies of LP neutrophils were also similar in OMM^12^ and OMM^12^ + MC^2^ mice (Fig. S1E). Conversely, neutrophils isolated from the intraepithelial compartment were markedly reduced in GF mice on days 8 and 10 p.i. compared to OMM^12^, OMM^12^ + MC^2^, and SPF mice (Fig. 1H). Notably, the varying numbers of intestinal neutrophils were not related to differences in numbers of blood neutrophils, as these were independent of the intestinal microbiota (Fig. S1F). Collectively, these data suggest a microbiota-triggered mechanism for neutrophil recruitment into the colonic lumen during *C. rodentium* infection.

### Commensals activate the intestinal endothelium

**Fig. 3:**
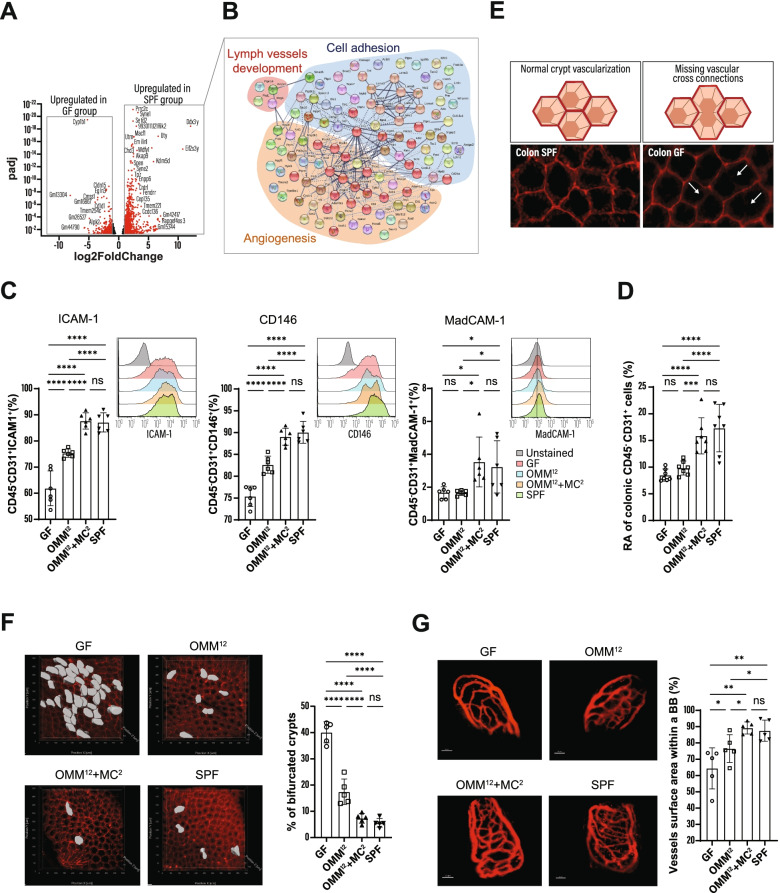
Commensals activate endothelial cells and promote intestinal angiogenesis. **A** Volcano plot showing fold change of gene expression in colonic endothelial cells of GF and SPF mice. Significant differences in gene expression between the two groups (log2-fold change ≥ 1; *p*-adj < 0.05) are marked in red. **B** STRING analysis for significantly upregulated transcripts of colonic endothelial cells from SPF mice. **C** Expression of ICAM1^+^, CD146^+^, and MadCAM1^+^ on colonic endothelial cells in indicated mice. **D** Relative abundance (RA) of colonic endothelial cells in the LP of indicated mice. **E** Schematic and microscopic representation of vascular cross connections in the colon. **F** Representative images and quantification of whole-mount staining indicated in mice. Partial vascularized crypts were quantified by insertion of contour surface modules (gray areas). **G** Intravital, multiphoton microscopy of small intestinal blood capillaries after i.v. injection of Qtracker™ 655. Vessel areas were quantified with trace image processing for morphometric measurements within a bounding box (BB). Scale bar: 30 μm. Results are representative of 4–5 experiments. Shown are representative data of at least two independent experiments with means ± SD; each symbol represents the individual value for one mouse (C-I). One-way ANOVA was used: ****p < 0.0001; **p ≤ 0.01; *p ≤ 0.05; ns, not significant

Extravasation of neutrophils requires the expression of LFA-1 on their surface and its interaction with ICAM-1 on endothelial cells for neutrophil arrest and diapedesis [21, 22]. During *C. rodentium* infection, high expression of LFA-1 was observed on blood and colonic neutrophils, independently of the host’s microbial status (Fig. S1G). In contrast, in steady state, the expression of ICAM-1 and CD146 on the vascular endothelium, both involved in cellular transmigration [23, 24], was influenced by the microbiota: low levels were observed in GF mice, while the expression in OMM^12^ + MC^2^ animals was similar to SPF mice (Fig. 3C). Although OMM^12^ colonization significantly enhanced endothelial ICAM-1 and CD146, expression levels similar to those in SPF mice were only achieved when the OMM^12^ consortium was enriched with *E. coli* and *C. amalonaticus* (MC^2^) (Fig. 3C). Similarly, normal amounts of the mucosal addressin MadCAM-1 [25] were only induced after colonization with the OMM^12^ + MC^2^ (Fig. 3C). The impact of the microbiota on activation of endothelial cells was also seen by transcriptome- and gene enrichment analysis (Fig. 3A and B). STRING analysis of significantly upregulated genes in SPF mice compared to GF mice revealed gene networks for cell adhesion, angiogenesis, and lymph vessel development in SPF mice (Fig. 3B). RNA-seq and subsequent pathway analysis revealed that 49 genes involved in angiogenesis were upregulated in colonic endothelial cells from SPF mice (Fig. 3B). In support of this data, microarray analysis of colonic endothelial cells from OMM^12^ and OMM^12^ + MC^2^ mice disclosed the upregulation of genes that are associated with angiogenesis in OMM^12^ + MC^2^ mice (Fig. S2A).

Having observed these angiogenic effects, the colonic tissue was investigated after whole-mount staining by 2-photon microscopy. Similar to previous studies [26], we noticed significantly increased branched crypts in the colon of GF mice (Fig. 3F). While OMM^12^ bacteria significantly reduced the numbers of bifurcated crypts, fully separated crypts typical for the mature colon [27] were only seen in OMM^12+^ + MC^2^ animals (Figs. 3F and 2). Importantly, staining of colonic tissue for CD31^+^ blood vessels revealed that reduced crypt bifurcations were accompanied by an increased number of vascular cross connections between crypts (Fig. 3E). In accordance with these observations, the relative abundance of endothelial cells was higher in OMM^12^ + MC^2^ and SPF mice (Fig. 3D). Additionally, we also observed colon size reduction and crypt lengthening after OMM^12^ and OMM^12^ + MC^2^ colonization (Fig. S2 C and D).

Previous studies have shown that permanent or transient colonization with *Bacteroides thetaiotaomicron* or *E. coli*, respectively, increased the microvascular density of the small intestine [28, 29]. Intravital microscopy revealed increased ramification of the villous vascular network in mice harboring the OMM^12^ + MC^2^ bacteria similar to SPF animals (Fig. 3G). The microvascular density of OMM^12^ mice was slightly increased as compared to GF mice, indicating that bacterial strains present in the OMM^12^ consortium have a limited potential to induce angiogenesis in the small intestine. Importantly, OMM^12^ + MC^2^ but not OMM^12^ consortia triggered ileal VEGFa expression (Fig. S2B).

Collectively, these results indicate that specific commensal bacterial strains activate the intestinal endothelium and promote angiogenesis to enhance leukocyte extravasation in the colon.

### Commensal bacteria initiate gene expression of vascular and immune networks in the adult intestine

**Fig. 4:**
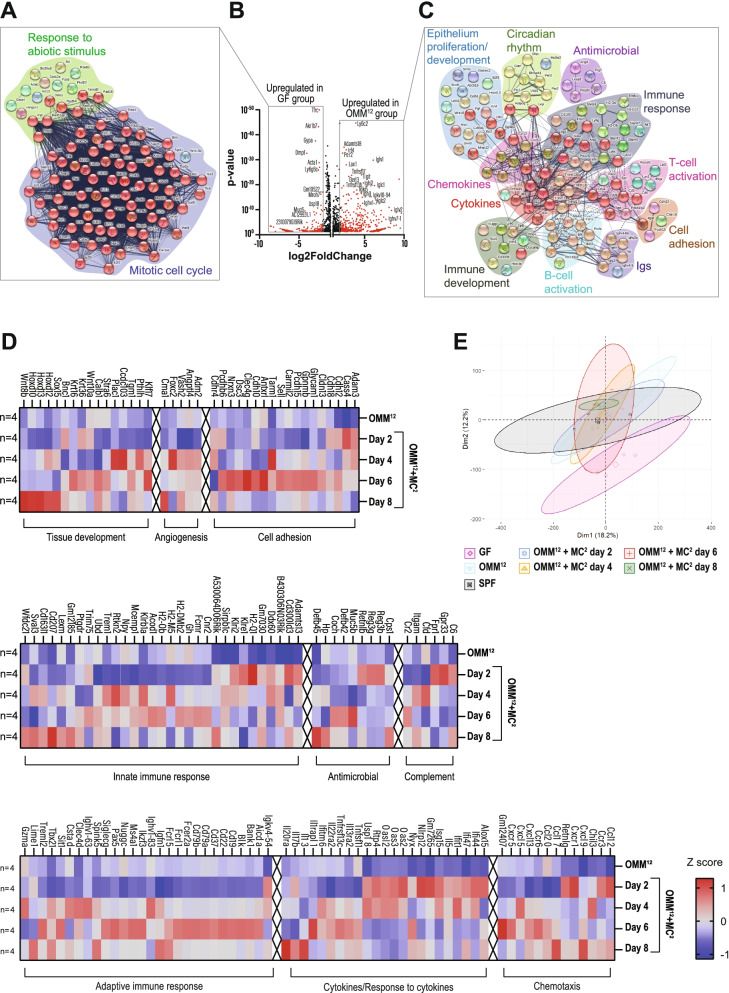
Commensal bacteria initiate gene expression of vascular and immune networks. **A**–**C** Volcano plot and STRING analysis showing fold change of gene expression in whole colon of GF and OMM^12^ mice (**B**). STRING analysis for significantly upregulated transcripts in the colon of GF (**A**) and OMM^12^ mice (**C**). **D** Heat map of differentially expressed gene clusters in OMM^12^ and OMM^12^ + MC^2^ at indicated time points after MC^2^ addition, log2-fold change ≥ 1; *p*-adj < 0.05. *n* = 4 biological replicates per group. **E** Principal component analysis of colonic gene expression in differently colonized mice. The axes correspond to principal component 1 (x-axis) and 2 (y-axis)

The cellular and morphological alterations triggered by commensals prompted transcriptome analysis of the whole colon from GF, gnotobiotic, and SPF mice. Animals colonized with OMM^12^ or OMM^12^ + MC^2^ revealed a considerably overlapping expression profile with SPF mice that was completely distinct from GF animals (Fig. 4E). To visualize differentially expressed transcripts, we first performed a two-group comparison between GF and OMM^12^ mice (Fig. 4A–C), which showed upregulated gene networks for innate and adaptive immunity, antimicrobial response, cell adhesion, and epithelial proliferation in OMM^12^ mice (Fig. 4C). In contrast, GF mice showed increased transcription of genes involved in the mitotic cell cycle and responses to abiotic stimuli (Fig. 4A). As the addition of MC^2^ to OMM^12^ was essential for the clearance of *C. rodentium*, the impact of the two commensals on host gene expression was studied. Gene network analysis between OMM^12^ and OMM^12^ + MC^2^ indicated that the presence of MC^2^ further amplified the host innate and adaptive immunity but also genes of the antimicrobial response and angiogenesis, e.g., angiopoietin-like 4, the ADM-RAMP2 system, VASH2, Reg3b, and Reg3g (Figs. 4D and S2F).

Interestingly, despite the broad imprinting effects of OMM^12^ and MC^2^ bacteria, FISH analysis revealed that this consortium did not invade the colonic epithelium (Fig. S2E).

Together, these findings illustrate that colonization of adult mice with limited numbers of commensal strains was sufficient to induce extensive mucosal reprogramming, comprising the maturation of the intestinal immune and vascular system as well as tissue development.

### Contribution of E. coli or C. amalonaticus to render OMM^12^ mice immunocompetent

**Fig. 5:**
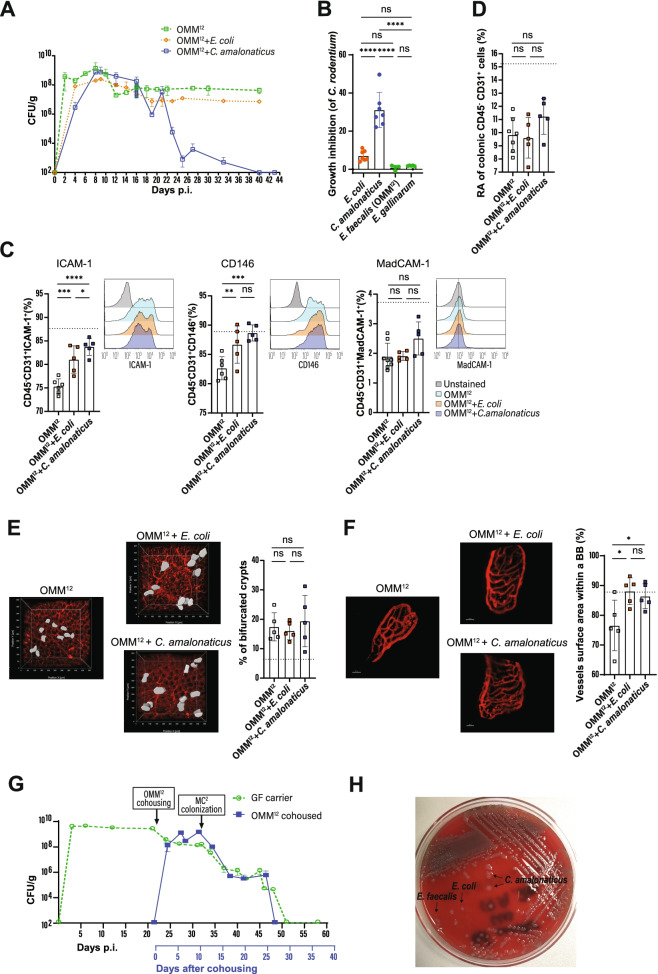
Impact of *E. coli* and *C. amalonaticus* on intestinal maturation and colonization resistance. **A** Course of *C. rodentium* infection in indicated mice. **B** In vitro growth inhibition of *C. rodentium* in the presence of indicated bacteria. Data are shown as fold change compared to *C. rodentium* O/N culture control. **C** Expression of ICAM1^+^, CD146^+^, and MadCAM1^+^ on colonic endothelial cells. **D** Relative abundance (RA) of colonic LP CD45^−^ CD31^+^ endothelial cells in colonized mice. **E** Representative quantification of vascularization in stained whole-mount tissues in indicated mice. Partial vascularized crypts were quantified by insertion of contour surface modules (gray areas). **F** Intravital, multiphoton microscopy of small intestinal blood capillaries after i.v. injection of Qtracker™ 655. Vessel areas were quantified with trace image processing to make morphometric measurements within a bounding box (BB). Scale bar: 30 μm. Results are representative of 4–5 experiments. **G** *C. rodentium* carriers were cohoused with OMM^12^ on day 22 p.i. All mice were gavaged 10 days later with MC^2^ bacteria. CFUs of *C. rodentium* were determined in feces. **H** Fecal colonies of *E.coli*, *E.faecalis* (*from OMM*^*12*^) and *C.amalonaticus* on Columbia blood agar universal plate from mice of experiment (**G**) after pathogen elimination. Animals were orally infected with (10^9^ CFU/ml) *C. rodentium*. Data shown are mean ± SD of 2–3 independent experiments; each symbol represents the value for one mouse (**D**–**F**). Dotted line represents the reference range for OMM^12^ + MC^2^ mice (**D**–**F**). One-way ANOVA was used: ****p < 0.0001; **p ≤ 0.01; *p ≤ 0.05; ns, not significant

As the composition and function of microbial communities are shaped by competitive and cooperative interactions among the constituent microbes [30, 31], we next investigated the impact of *E. coli* and/or *C. amalonaticus* on *C. rodentium* elimination in the presence or absence of OMM^12^. We noted a significantly reduced pathogen burden after associating OMM^12^ mice with *E. coli* in comparison with OMM^12^. However, pathogen clearance was not achieved (Fig. 5A). In contrast, OMM^12^ + *C. amalonaticus* colonization was sufficient to mediate delayed pathogen clearance after 6 weeks (Fig. 5A), compared to 3 weeks in SPF and OMM^12^ + MC^2^ mice (Fig. 1D). As microbial competition for space and resources may have been responsible for these effects, the in vitro potential of *E. coli* and *C. amalonaticus* to compete against *C. rodentium* was studied. While *C. amalonaticus* strongly inhibited the growth of *C. rodentium*, *E. coli* failed to do so comparable to *Enterococcus faecalis* from the OMM^12^ and *E. gallinarum* from the MC^3^ consortium (Fig. 5B). Interestingly, in monocolonized mice, *E. coli*, as previously reported [14], as well as *C. amalonaticus* decreased the *C. rodentium* burden to similar extents, demonstrating that competition mechanisms differ in vitro and in vivo (Figs. 5B and S3A). Colonization of GF mice with both bacteria (MC^2^) resulted in enhanced pathogen colonization resistance, indicating that both commensals cooperate in vivo (Fig. S3A).

Next, the influence of OMM^12^ + *E. coli* and OMM^12^ + *C. amalonaticus* on the expression of cell adhesion molecules in endothelial cells was examined. Both groups showed significantly increased ICAM-1 and CD146 expression compared to OMM^12^ alone, although *C. amalonaticus* induced a slightly stronger ICAM-1 expression than *E. coli* in the presence of OMM^12^ (Fig. 5C). The expression of MadCAM-1, however, was not affected by either commensals (Fig. 5C). Importantly, monocolonization with *E. coli* or *C. amalonaticus* induced similar effects but to a lesser extent (Fig. S3B). Although in the presence of OMM^12^ both bacteria induced strong expression of CD146, a marker for angiogenesis [24], the number of endothelial cells was not increased (Fig. 5D). In agreement with this, enrichment of OMM^12^ mice with either *E. coli* or *C. amalonaticus* did not result in crypt maturation and formation of vascular cross connections in the colon, as seen after OMM^12^ + MC^2^ colonization (Fig. 5E). On the other hand, monocolonization with *E. coli* or *C. amalonaticus* led to a reduction of bifurcated crypts in ex-GF mice (Fig. S3D), but the relative abundance of endothelial cells remained the same as in GF mice (Fig. S3C). Notably, the relative abundance of endothelial cells increased only once the percentages of bifurcated crypts were approx. 5%, which was only seen in OMM^12^ + MC^2^ and SPF mice (Fig. 3F). As the medianl colon was investigated, we cannot exclude that crypt bifurcations in the proximal or distal colon are also affected by microbial colonization. Conversely, intravital microscopy of the small intestine revealed that monocolonization with *E. coli* or *C. amalonaticus*, or in the presence of OMM^12^, increased the density of the capillary network in villi (Figs. 5F and S3E).

Finally, the therapeutic effect of the OMM^12^ + MC^2^ consortium in long-term *C. rodentium*-carrier GF mice was examined. GF carriers were first cohoused with OMM^12^ mice and subsequently colonized with MC^2^ bacteria (Fig. 5G). Cohousing with OMM^12^ mice led to a 200-fold reduction of the fecal pathogen count in the GF carriers, while the OMM^12^ cohoused mice became quickly infected with *C. rodentium* (Fig. 5G). Ten days after OMM^12^ cohousing, all mice received MC^2^ bacteria, which resulted in a continuous pathogen decrease until its elimination (Fig. 5G). It is noteworthy that both *E. coli* and *C. amalonaticus* stably colonized the animals and were detectable after pathogen clearance (Fig. 5H).

These findings illustrate that the OMM^12^ consortium serves as a basis for MC^2^ bacteria to better exert its imprinting effects on the host. Moreover, the cooperation of these 14 bacteria is required for the conversion of *C. rodentium* carriers into responders. In this case, the MC^2^ consortium represents two commensals that compensate for partial defects in the microbiota and thus restore immunity via maturation and activation of the gut vascular system.

## Discussion

In this study, we show that selected 14 bacterial strains stably colonize the gut of adult GF mice and stimulate morphological and immunological maturation of the gut. Although the 12 bacteria of the OMM^12^ community activated many immune parameters, they were not able to eliminate *C. rodentium*, demonstrating that the presence of two commensals is critical for full immunocompetence. Clearance of *C. rodentium* has been shown to require at least two major steps: first, killing of antibody-coated virulent bacteria by neutrophils in the intestinal lumen, and secondly, metabolic competition and colonization resistance of avirulent bacteria by commensals [14, 19, 20]. Our results revealed that the number and frequencies of blood and colonic LP neutrophils are independent of the microbial status of the host during *C. rodentium* infection, but not those that transmigrate through the colonic epithelium. Here, association of GF animals with OMM^12^ + MC^2^ triggered strong migration of neutrophils into the colonic lumen for pathogen engulfment. Thus, trans-endothelial migration of neutrophils into the gut can be modulated by microbial signals. Notably, while OMM^12^ colonization induced robust neutrophilic migration during the infection, this was not sufficient to trigger the complete elimination of *C. rodentium*. Since facultative anaerobes are underrepresented in the OMM^12^ consortium [16], we propose that MC^2^ bacteria provided bacterial competition which inhibited the expansion of avirulent *C. rodentium* and together with colonic neutrophils led to pathogen clearance. Of note, although *C. rodentium* was avirulent in long-term GF-carrier mice, they were able to spread the pathogen and infect naïve animals, similar to human carriers. It was previously shown that a bicarbonate-rich environment, such as the culture medium DMEM or the gastrointestinal tract, activates *C. rodentium* virulence genes to restore the infectivity of previously avirulent bacteria [32–34].

As composition and function of microbial communities are shaped by competitive and cooperative interactions among the constituent microbes [30, 31], we also dissected the impact of *E. coli* and *C. amalonaticus* during the enteric infection. *C. amalonaticus* in combination with the OMM^12^ community led to a substantial delayed but effective clearance of *C. rodentium* infection. This finding supports the observation that SPF mice harboring *C. amalonaticus* show strong colonization resistance against *C. rodentium* infections [18]. In contrast, addition of *E.coli* to OMM^12^ mice failed to eliminate the pathogen but showed marginal reduction of the pathogen burden, as previously reported in monocolonized mice [14]. The fundamental role of the OMM^12^ consortium to support MC^2^ bacteria in the progression of intestinal maturation and pathogen clearance is demonstrated in *E. coli* and *C. amalonaticus* monocolonized mice. The only effect shared between all bacterial consortia combinations and OMM^12^ + MC^2^ was small intestinal angiogenesis, thus highlighting the complex microbial cooperation between the 14 commensals.

Diapedesis requires the interaction of neutrophil-expressed LFA-1 with ICAM-1, which is expressed on endothelial cells that form the inner cellular lining of blood and lymphatic vessels [22, 35]. Gene analysis of colonic endothelial cells from SPF and OMM^12^ + MC^2^ mice revealed that gut commensals promote their activation and proliferation. Remarkably, OMM^12^ colonization of GF mice increased the expression of ICAM-1 and CD146 on colonic endothelial cells, but normal levels were only achieved in the presence of MC^2^. Of particular importance was the observation that the OMM^12^ + MC^2^ community triggered the development of the villous capillary network in the intestine and induced maturation of the colon via a process called crypt fission [36]. Crypt fission is a developmental process that primarily occurs during the postnatal period and to a much lesser extent in adults [37].

Enrichment of the OMM^12^ community with MC^2^ resulted in transcriptional upregulation of several genes expressed in epithelial and/or endothelial cells that protect intestinal stem and Paneth cells from apoptosis and thus contribute to the development of blood vessels, vascular integrity, and homeostasis [38–42]. The Wnt signaling pathway plays a key role in the maintenance and progenitor proliferation of intestinal stem cells, the maturation of Paneth cells, and expression of cryptdins [43, 44]. In line with this, parathyroid hormone-related protein (PTHrP) was upregulated in the colon of OMM^12^ + MC^2^, a factor involved in the activation of the Wnt signaling and the regulation of angiogenesis via VEGF expression [45]. Here, VEGF expression was detected in the ileum of OMM^12^ + MC^2^ but not OMM^12^, demonstrating that cooperation with MC^2^ is essential for triggering angiogenesis in the small intestine and colon.

Finally, an aspect of clinical importance is that the functional interaction between OMM^12^ and MC^2^ also works after successive colonization, indicating that preexisting microbiota can be corrected or complemented. Whether the mechanisms of these microbe-triggered angio- and immunogenic activities involve direct interactions of bacterial ligands such as TLR- or NOD-like receptors [46] and/or indirect ones via metabolites [47, 48] remain to be elucidated, but we are convinced that the observed effects are not limited to the microbial strains used in this study. Although the understanding of commensal-induced tissue and immune-development is complex, our findings give new insights into commensal/microbe-triggered host functionalities. This may be of therapeutic use not only for treatment of asymptomatic carriers of enteropathogens [49] but also for providing an adequate immune response against various assaults [50] or for promoting immune-responsiveness in immunotherapy-refractory patients [51, 52].

In summary, selected commensal bacteria are able to reinitiate ontogeny-like gene expression in the adult intestine, inducing morphological and immunological maturation required for the elimination of enteropathogens.

## Conclusions

Our findings revealed that colonization of an enteropathogen carrier with a selected bacterial consortium initiated pathogen elimination. Carrier to responder conversion was made possible through microbe-induced maturation and activation of the intestinal vascular and immune system. Different bacterial consortia have been shown to be of therapeutic value with regard to anticancer immunity [53], chronic infections [54, 55], inflammation [56], or the conversion of immunotherapy-refractory patients into responders [57]. Therefore, we believe that the here observed effects are not exclusively limited to the OMM^12^ + MC^2^ community. However, integration and stable colonization of specific bacteria into an existing microbiota are essential prerequisites for microbe-based therapies. Still, intensive research is required to determine the molecular mechanisms by which such microbial consortia act.

In summary, selected commensals are able to reinitiate ontogeny-like gene expression in the adult intestine, thus triggering morphological and immunological maturation, both essential for the elimination of enteropathogens.

## Material and methods

### Mice

All mice were kept in the animal facility of the Biomedical Research Centre of Philipps-University Marburg. WT mice (C57B/6 background) were purchased from the Charles River Laboratory and bred under specific pathogen-free (SPF) conditions in individually ventilated cages (IVC), with 12-h light cycle, standard rodent pellet diet (LasQCdiet Rod18, HiHyg (Altromin)), and water ad libitum. Germ-free (GF) mice harboring the OMM^12^ minimal consortium (C57B/6 background) were kindly provided by Dr. M. Basic (Hannover). GF and gnotobiotic mice were bred in sterile isolators with positive pressure differential and filter-top cages (Tecniplast) with autoclaved bedding, food (Altromin 1324 P Forti), and water ad libitum. Sterility in GF mice was checked monthly by culturing feces in thioglycollate medium under aerobic and anaerobic conditions for at least 10 days as well as fecal gram staining. Contamination in gnotobiotic mice was checked by culturing the feces on blood agar plates under aerobic conditions. All handling procedures for GF and gnotobiotic mice were conducted under a laminar flow hood under sterile conditions.

### C. rodentium infection

The nalidixic acid (NA)-resistant wild-type *Citrobacter rodentium* strain DBS 100 was a gift from Till Strowig. For inoculation, bacteria were grown in Luria-Bertani (LB) broth, supplemented with NA (50 μg/ mL) overnight with shaking at 37 °C. Mice were orally infected with 0.2 mL of a *C. rodentium*-PBS suspension (10^9^ CFUs/ml). Bacterial titer was quantified in stool samples by performing serial dilutions, plating bacterial suspensions on LB agar containing NA (50 μg/ mL), and incubating plates overnight at 37 °C.

### mRNA isolation and qRT-PCR

For total RNA extraction, TRI Reagent (Sigma-Aldrich) was used. RevertAid First Strand cDNA Synthesis Kit (Thermo Scientific) was used to generate complementary DNA (cDNA) according to the manufacturer’s instructions. For qRT-PCR, SYBR Green (Applied Biosystems) was applied and the following primer pairs were used:

- *ler*Fwd: ACA ACA AGC CCA TAC ATT CAG C
  Rev: TGT TAC TTC TTC TTC TGT GTC CTT CA
- *map* Fwd: CGG CTA CAC AAA CTC TTA GAC CAG
  Rev: CTT TAC CGC ACT GCT CAT CAAC
- *espG* Fwd: GTG GCG ATT GAT GGG TAA AGAT
  Rev: AAA AGC CGT GGA ATG AGA TGAC
- *espJ* Fwd: ATG CTT TTG GTA TCA CTA CGG G
  Rev: ATG GGT ATA TGT CAA CAT CCA GTC T
- *16S* Fwd: GGT TGG TGC CTT CGG GAA CTC
  Rev: CGC GAG GTC GCT TCT CTT TG

Quantification of cDNA was carried out by normalization to expression of the housekeeping gene 16S using the ΔΔCt method.

### Application of RB6-8C5 and NIMP-R14 neutrophil-depleting antibodies

RB6-8C5 and NIMP-R14 antibodies were kindly provided by Dr. Friderike Jönsson, from the Institute Pasteur in Paris. They were diluted in PBS and applied i.p. (500 μg/injection) on the days 5, 8, 11, 14, and 16 p.i. to each mouse.

### Fecal microbiota transplant (FMT) of GF mice

Feces from the caecum and colon of SPF mice were homogenized in PBS and given to GF mice infected with *C. rodentium* on day 3 p.i.

### Aerobic bacterial suspension from SPF stool samples and bacterial isolation

SPF stool contents from ceacum and colon were transferred to an Erlenmeyer flask containing 100 ml LB medium. The suspension was incubated overnight (~16–18 h) at 37 °C. A total of 0.2 ml of the suspension was given to GF mice infected with *C. rodentium* (day 22 p.i.). After pathogen elimination, stool samples from mice that received the aerobic bacteria suspension were homogenized with PBS and streaked onto different agar plates (Columbia-blood agar, chocolate, *Clostridium difficile*, Müeller-Hinton, and Schaedler agar) and incubated under different conditions (aerobic, anaerobic, and CO_2_) at 37 °C overnight or until colonies were formed. Colonies with different morphologies were isolated and streaked onto new plates. The identification of the different isolated bacterial strains was done using matrix-assisted laser desorption ionization time-of-flight mass spectrometry (MALDI-TOF MS).

### Sequence analyses of E.coli and C. rodentium

In short, bacterial genomic DNA was extracted using the PureLink Genomic DNA Mini Kit (Invitrogen) as recommended by the manufacturer. For genome sequencing Illumina short-read sequencing technology and for long-read sequencing, Oxford Nanopore Technology was used. Detailed genome sequencing is given as supplemental information, extended bacteria sequencing.

Briefly, the genome of *Escherichia coli* RSHH22 consists of a circular chromosome of 5,094,340 bp and two extrachromosomal circular sequences designated as plasmid pEC_RSHH22_1 (161,535 bp) and plasmid pEC_RSHH22_2 (4538 bp). The genome harbors 5169 genes in total. We identified eight 5S and seven 16S and 23S rRNA genes. Eighty-nine tRNA genes were detected. The genome of *Citrobacter amalonaticus* RSHH22 consists of a circular chromosome of 4,877,251 bp and one extrachromosomal circular sequence designated as plasmid pCA_RSHH22_1 (110,958 bp). The genome harbors 4808 genes in total. For *Citrobacter amalonaticus* RSHH22, we identified eight 5S and seven 16S and 23S rRNA genes. Eighty-nine tRNA genes were detected. Genome sequence data for *E. coli* RSHH22 and *C. amalonaticus* RSHH22 have been submitted to the NCBI under the accession numbers CP096902-CP096904 and CP096905-CP096906. Potential virulence factor genes and antibiotic resistance genes can be found in supplemental Table 1.

### Association of mice with commensal bacteria

A single colony from the isolated bacterial strains was inoculated in 50 ml LB medium and incubated at 150 rpm at 37 °C overnight. The overnight culture was centrifuge (20 min, 4000 rpm at 4 °C). The pellets were resuspended in PBS and mix together as follows:

- MC^5^: *L. murinus* (1:4), *E. gallinarum* (1:4), *Paenibacillus* sp. (1:4), *E. coli* (1:8), and *C. amalonaticus* (1:8)
- MC^3^: *L. murinus* (1:3), *E. gallinarum* (1:3), and *Paenibacillus* sp. (1:3)
- MC^2^: *E. coli* (1:1) and *C. amalonaticus* (1:1)

Mice were orally gavage with 0.2 mL of the bacterial-PBS suspension.

### Cell isolation techniques

Single-cell suspensions were performed from the intestine by mechanical disruption and passage through filter (Miltenyi Biotec). Lamina propria cells and intraepithelial cells were isolated using the Lamina Propria Dissociation Kit (Miltenyi Biotec) according to manufacturer’s instructions. In short, tissues were transferred to preheated digestion solution in C tubes (Miltenyi Biotec) and processed by gentleMACS Octo Dissociator (Miltenyi Biotec). The obtained cell suspension was filtered on a 100 μm cell strainer and washed with MACS buffer. Cells were centrifuged (300 × g, 4 °C, 10 min) and counted using the TC20 automated cell counter (Bio-Rad). Leukocytes were depleted or isolated by incubating the cell suspension with CD45 beads (Miltenyi Biotec). For endothelial activation, the CD45-negative lamina propria cells were further isolated using CD31 beads (Miltenyi Biotec).

### Whole blood isolation

Mice were euthanized with isoflurane, and blood was drawn through cardiac puncture. Blood was collected in 2-ml Eppi tubes with HBSS prep supplemented with 80 μL of heparin solution (25000 I.U./5 ml). Blood was pipetted on top of 3 ml Histopaque 119 (Sigma) in a 15-ml falcon tube to obtain a density gradient. Samples were centrifuged at RT for 5 min at 300 g followed by 20 min at 800 g without break. Interphase containing white blood cells was pipetted into a new 15 falcon tube and wash with FACS buffer. Pellets were treated for erythrocyte lysis with 5 ml NH4CL solution for 3 min at RT. Cells were centrifuged (300 × g, 4 °C, 10 min) and counted using the TC20 automated cell counter (Bio-Rad).

### Cell staining procedures and flow cytometry

Prior to staining for cell surface markers, cell suspension was incubated with FcR Blocking Reagent (Miltenyi) for 10 min at 4 °C. Cells were stained by incubation with antibodies diluted in PBS/1%FCS/2 mM EDTA (FACS) buffer for 15 min at 4 °C. After further washing with FACS buffer, cells were fixed in 2% formaldehyde for 20 min at 4 °C. Flow cytometric analysis was performed after further washing steps, using Attune NxT Flow Cytometer (Thermo Fisher).

For RNA sequencing, CD45^−^CD31^+^ICAM-1^+^CD146^+^ cells were sorted on FACSAria™ III (BD Biosciences).

### Whole-mount staining of colonic tissue

Mice were euthanized with isoflurane and perfused with ice-cold PBS containing heparin. Colon was cut longitudinally, and feces were washed out. The tissues were placed in a 12-well plate containing paraformaldehyde (PFA) solution as fixative reagent. After 2 h at 4 °C, the samples were washed 3 times (30 min at 4 °C) with PBS with 0.3% Triton X. Tissues were incubated for 2 h at RT in 10% normal goat serum (NGS) containing 0.2% Triton-X. Vessels were stained with Alexa Fluor® 594 anti-mouse CD31 antibody (Biolegend) in 2% NGS and incubated for ca. 60 h at 4 °C. After antibody incubation, the immunolabeled samples were washed in PBS overnight and transferred into the bracket of the autostainer. The first vial contained a 50% EtOH solution with Aqua ad Iniectabilia. For adjusting the pH value, 0.1 M NaOH and 0.1 M HCl were used. The samples were placed into an ascending ethanol order, starting from 50 to 70% and two times 100% EtOH, to slowly dehydrate the tissue. Each dehydration step was performed for 4 h. After dehydration, the sample was played into a glass vessel and incubated with ethyl cinnamate (ECi) (Sigma). After at least 2 h incubation in refractive index (RI) matching agent, the translucent sample was put into a modified view chamber containing ECi and was analyzed using a 2-photon microscope (Olympus FVMPE-RS). For quantification, up to 200 crypts per individual image were examined. Using Imaris surface modules for marking crypts partially surrounded by vascular vessels, the vascularization of crypts was quantified.

### Intravital microscopy

Mice were anesthetized by intraperitoneally injection of ketamine (0.1 g/kg BW) and 0.01 g xylazine (0.01 g/kg BW) (injection solution: 10 μl/g BW). Mice were placed on a preheated plate at 37 °C, and the depth of anesthesia was monitored by checking withdrawal reflexes and using the MouseSTAT® Jr (Kent Scientific) heart monitor. A small piece of the ileum (~1 cm) was taken, longitudinally open, feces were flushed out, and the tissue was fixed on a preheated metal plate using agarose gel and tissue glue. After preparation of the ileum, mice were injected intravenously with Qtracker™ 655 vascular labels (Invitrogen) to provide real-time vascular contrast. Image acquisition was done using a 2-photon microscope (Olympus FVMPE-RS). Afterwards, mice were euthanized. Quantification was made using Imaris Stitcher and Imaris software (Bitplane) to manually construct surface modules from the fluorescence intensity. The surface area was measure within a surface bounding box area.

### Immunohistochemistry VEGF

For immunohistochemistry, heat-induced epitope retrieval was performed with EDTA. Staining was performed on a DAKO autostainer plus. After blocking endogene peroxidase, sections were incubated for 45 min with mouse monoclonal anti-VEGFA antibody (1:200; Abcam no. ab1316, clone VG-1). Sections were washed and incubated with Dako REAL EnVision HRP Rabbit/Mouse polymer, which reacts with DAB chromogen, according to the manufacturer’s protocol.

### Transcriptional profiling

#### RNA-seq of endothelial cells

For RNA-sequencing of endothelial cells, sorted endothelial cells were resuspended in RLT-buffer containing b-mercaptoethanol. RNA was purified using the RNeasy Plus Micro Kit (Qiagen) according to the manufacturer’s instructions. RNA was quantified with a Qubit 2.0 fluorometer (Invitrogen), and the quality was assessed on a Bioanalyzer 2100 (Agilent) using a RNA 6000 Pico chip (Agilent). Samples with an RNA integrity number (RIN) of > 8 were used for library preparation. Barcoded mRNA-seq cDNA libraries were prepared from 20 ng of total RNA using NEBNext® Poly(A) mRNA Magnetic Isolation Module and NEBNext® Ultra™ II RNA Library Prep Kit for Illumina® according to the manual with a final amplification of 15 PCR cycles. Quantity was assessed using Invitrogen’s Qubit HS assay kit, and library size was determined using Agilent’s 2100 Bioanalyzer HS DNA assay. Barcoded RNA-Seq libraries were onboard clustered using HiSeq® Rapid SR Cluster Kit v2 using 8 pM, and 59 bps were sequenced on the Illumina HiSeq2500 using HiSeq® Rapid SBS Kit v2 (59 cycles). The raw output data of the HiSeq was preprocessed according to the Illumina standard protocol. Sequence reads were trimmed for adapter sequences and further processed using Qiagen’s software CLC Genomics Workbench (v20 with CLC’s default settings for RNA-Seq analysis). Reads were aligned to GRCm38 genome. Total read counts were further processed in R using the DeSeq2 package to compute differential gene expression and adjusted *p*-values. Gene ontology (GO) analysis and pathway enrichment were performed using PANTHER and AmiGO. Known and predicted interactions for differentially regulated genes (log2fold change higher than 1 and adjusted *p*-value lower than 0.05) were derived using the STRING database. The RNA-Seq data were deposited in the Gene Expression Omnibus (GEO) of the National Center for Biotechnology Information and can be accessed with the GEO accession number GSE 180156 (https://www.ncbi.nlm.nih.gov/geo/query/acc.cgi?acc=GSE180156).

### RNA-seq of whole colon

For whole colon RNA sequencing, colon tissues were preserved directly after dissection in Allprotect Tissue Reagent (Qiagen). Total RNA was isolated using AllPrep DNA/RNA/Protein Mini Kit (Qiagen) according to the manufacturer’s instructions. The RNA was treated with RNase-free DNase set (Qiagen cat no.: 79254) and converted to cDNA synthesis technology and generates Illumina-compatible libraries via PCR amplification (Takara cat no.: 634444). The directionality of the template-switching reaction preserves the strand orientation of the original RNA according to the manufacturer’s protocol. Libraries were sequenced for PE100 cycles to a depth of 30 million paired reads using Illumina NovaSeq 6000. Overrepresentation analysis (ORA) and Gene Set Enrichment Analysis (GSEA) were performed for significant differentially expressed protein-coding up- and downregulated genes from RNA-Seq data. Gene Ontology ORA was done using enrichGO method from clusterProfiler. Correlation analysis was performed for normalized counts of all the protein-coding genes from all the samples. The RNA-Seq data were deposited in the Gene Expression Omnibus (GEO) of the National Center for Biotechnology Information and can be accessed with the GEO accession number GSE 181502 (https://www.ncbi.nlm.nih.gov/geo/query/acc.cgi?acc=GSE181502).

### Microarray analysis

For microarray analysis of endothelial cells, MACS-isolated endothelial cells were resuspended in RLT buffer containing b-mercaptoethanol. RNA was purified using the RNeasy Plus Mini Kit (Qiagen) according to the manufacturer’s instructions. RNA quantity and quality were assessed with an Experion StdSens Chip on an Experion™ Automated Electrophoresis System (Bio-Rad). Microarray experiments were performed as dual-color dye-reversal color-swap hybridizations. Total RNA was labeled with the Low Input Quick-Amp Labeling Kit (Agilent Technologies). In brief, 100 ng total RNA was reverse transcribed and amplified using an oligo-dT-T7 promoter primer and labeled with cyanine 3-CTP and/or cyanine 5-CTP by T7 in vitro transcription. After precipitation, purification, and quantification, 500 ng labeled cRNA of each ratio sample was mixed, fragmented, and hybridized to SurePrint G3 Mouse GE v2 8 × 60 K multipack microarrays (Agilent-074809) according to the supplier’s protocol (Agilent Technologies). Scanning of microarrays was performed with 3 μm resolution and 20-bit image depth using a G2565CA high-resolution laser microarray scanner (Agilent Technologies). Microarray image data were processed with the Image Analysis/Feature Extraction software G2567AA v. A.12.1.1.1 (Agilent Technologies) using default settings and the GE2_1200_Dec17 extraction protocol. The extracted MAGE-ML files were analyzed with Rosetta Resolver, Build 7.2.2 SP1.31 (Rosetta Biosoftware). Color-swap ratio profiles comprising single hybridizations were combined in an error-weighted fashion to create ratio experiments. A 0.5 log2-fold change expression cutoff for ratio experiments was applied together with anti-correlation of ratio profiles, rendering the microarray analysis highly significant (*p* < 0.05). In addition, microarray data was analyzed using the R package limma [Ritchie ME, Phipson B, Wu D et al. limma powers differential expression analyses for RNA-sequencing and microarray studies. Nucleic Acids Res. 2015;43(7):e47. doi:10.1093/nar/gkv007]. The microarray readout txt files were background corrected, normalized, and controlled for quality [Smyth, G. K., and Speed, T. P. (2003). Normalization of cDNA microarray data. Methods 31, 265{273] (version 3.40.6). Within-array normalization was done by locally estimated scatterplot smoothing (Loess), and between-array normalization was done using the Aquantile method. The hybridization control samples were removed, and the gene expression values were averaged for each probe over all replicates of that probe on the microarray, using the avereps function. Microarray data were deposited in the Gene Expression Omnibus (GEO) of the National Center for Biotechnology Information and can be accessed with the GEO accession number GSE 180907 (https://www.ncbi.nlm.nih.gov/geo/query/acc.cgi?acc=GSE180907).

STRING analysis for protein-protein interactions (highlighted and labelled) was performed on differentially expressed genes (log2-fold change > 1; p-adj < 0.05) showing biological processes related to upregulated genes. Line thickness indicates the strength of data supporting the connections between the proteins according to the STRING database. MCL clustering (inflation parameter = 3) was applied to cluster the networks, where every color corresponds to a cluster with similar or associated functions (known and predicted).

### Fluorescence in situ hybridization (FISH)

Colonic intestinal tissues containing fecal pellets were fixed overnight with methacarn at room temperature. Tissues were washed in methanol for 30 min, two times in ethanol for 15 min, once in ethanol/xylene (1:1) for 15 min, and two times in xylene for 15 min prior to paraffin embedding.

For FISH analysis, 3–5 μm thin tissue sections were dewaxed and then treated with 50 μl 4% lysozyme solution (45 min, 37 °C) for nucleic acids demasking. After washing, 50 μl hybridization solution (0.9M NaCL, 20 mM Tris-HCL, 0.05% SDS) with the bacterial probe (1:50 Eub338 FITC-GCT GCC TCC CGT AGG AGT) was added and incubated for 3 h at 50 °C. Slides were washed several times at 37 °C and were dried at RT before mounted with ProLong™ Gold Antifade Mountant with DAPI (Thermo Fisher Scientific) following manufacturer’s instructions. The samples were documented at a Leica DM5500 wide-field microscope (Leica) and analyzed with Imaris software (Bitplane).

### In vitro bacterial competition assay

Bacterial competition assay was performed as previously described with a few modifications [58]. In short, a single colony from *C. rodentium* was incubated in 100-LB medium with a single colony of another commensal bacterial strain overnight at 37 °C (150 rpm). Bacterial suspension was centrifuged, and the CFU/ml of *C. rodentium* was quantified using serial dilutions. Growth inhibition was measured as normalized fold changes to *C. rodentium* normal growth in LB medium.

### Statistics

The data were analyzed with GraphPad Prism 9. Significance was calculated using tests indicated in the figure legends. Values less than 0.05 were considered statistically significant.

## Supplementary Information

**Additional file 1: Figure S1.** Microbiota determines neutrophil mediated elimination of *C. rodentium*. (A) Fecal CFU in *C. rodentium* infected SPF and GF mice, after fecal microbiota transplant (FMT) from SPF mice. (B) *C. rodentium*-carriers were gavaged on day 22 p.i with an overnight (O/N) aerobic culture of feces from SPF animals, CFUs were determined. (C) Fecal CFUs of *C. rodentium* in indicated mice. (D) Kinetic of colonic neutrophils absolute cell numbers (ACN) during the course of *C. rodentium* infection. (E) Neutrophil frequencies in the colonic lamina propria of indicated mice at day 8 and10 p.i. (F) ACN of neutrophils in the blood of indicated mice at day 8 and 10 p.i. (G) Frequencies of LFA-1^+^ neutrophils in the blood and colon of *C. rodentium* infected mice. For *C. rodentium* infection: animals were orally infected with (10^9^ CFU/ml) *C. rodentium.* Data shown are means ±SD and representative of at least two independent experiments, each symbol represents the individual value for one mouse (G-I). One-way ANOVA was used: ****p < 0.0001; ** *p* ≤ 0.01; * *p* ≤ 0.05; ns, not significant. **Figure S2.** Commensal triggered gene expression and macroscopic changes of the colon. (A) Volcano plot showing fold-change of gene expression in colonic endothelial cells of OMM^12^ and OMM^12^ +MC^2^ (log2-fold change≥1; *p*-adj < 0.05). STRING analysis for significantly upregulated transcripts in colonic endothelial cells of OMM^12^ (left) and OMM^12^ + MC^2^ (right) mice. (B) Representative immunostaining and quantification of VEGFa expression (brown staining) in the ileum of indicated mice. (C) Macroscopic alterations and colon length in response to the microbiota. (D) Quantification of colonic crypt length in indicated mice, each symbol represents one crypt (*n*=3). (E) OMM^12^+MC^2^ bacteria were visualized by FISH in medial colon (red, bacteria; blue, DAPI). (F) STRING analysis of differentially expressed genes (log2-fold change ≥1; *p*-adj < 0.05) in complete colon of OMM^12^ after colonisation with MC^2^ (*n* = 4 replicates per group). For *C. rodentium* infection: animals were orally infected with (10^9^ CFU/ml) *C. rodentium.* Data shown are means ±SD; at least two independent experiments were performed, each symbol represents the individual value for one mouse (A, C-F). One-way ANOVA was used: ****p < 0.0001; ** *p* ≤ 0.01; * *p* ≤ 0.05; ns, not significant. **Figure S3.** Effects of GF mice monocolonization with *E. coli* or *C. amalonaticus*. (A) Fecal *C. rodentium* CFUs in GF and colonized mice after oral infection with *C. rodentium*. (B) Frequencies of ICAM1^+^, CD146^+^ and MadCAM1^+^ colonic endothelial cells in indicated animals. (C) Relative abundance (RA) of LP-isolated CD45^-^CD31^+^ colonic endothelial cells. (D) Representative images and quantification of whole mount staining in indicated mice. Partial vascularized crypts were quantified by insertion of contour surface modules (gray areas). (E) Intravital, multi-photon microscopy of small intestinal blood capillaries after i.v. injection of Qtracker™ 655. Vessel areas were quantified with trace image processing to make morphometric measurements within a bounding box (BB). Scale bar: 30μm. Results are representative of 4-5 experiments. Data represent mean ±SD of 2-3 independent experiments, each symbol represents the value for one mouse (A, B, D-F). Dotted line represents the reference range for OMM^12^ mice (D-G). One-way ANOVA was used: ****p < 0.0001; ** *p* ≤ 0.01; * *p* ≤ 0.05; ns, not significant. **Figure S4.** Gene networks from Fig. 4. STRING analysis for significantly upregulated transcripts in the colon of GF (A) and OMM^12^ mice (B).

**Additional file 2: Supplemental Table 1.** Results of virulence factor and antibiotic resistance gene prediction based on blastp&VFDB and RGI&CARD.

**Additional file 3.**

## Data Availability

All the data associated with this study are present in the paper or the supplementary materials.

## Abbreviations

ACN: Absolute cell number
BB: Bounding box
CFUs: Colony-forming units
EPEC: Enteropathogenic *E. coli*
EHEC: Enterohemorrhagic *E. coli*
GF: Germ-free
MC: Minimal consortium
LP: Lamina propria
OMM^12^: Oligo-Mouse-Microbiota
O/N: Overnight culture
RA: Relative abundance
SPF: Specific pathogen-free
