# Supplementary material for: Selected commensals educate the intestinal vascular and immune system for immunocompetence
Source: Microbiome. 2022 Sep 28;10:158. doi: 10.1186/s40168-022-01353-5 (PMC9520927; doi:10.1186/s40168-022-01353-5)

Supplemental 1

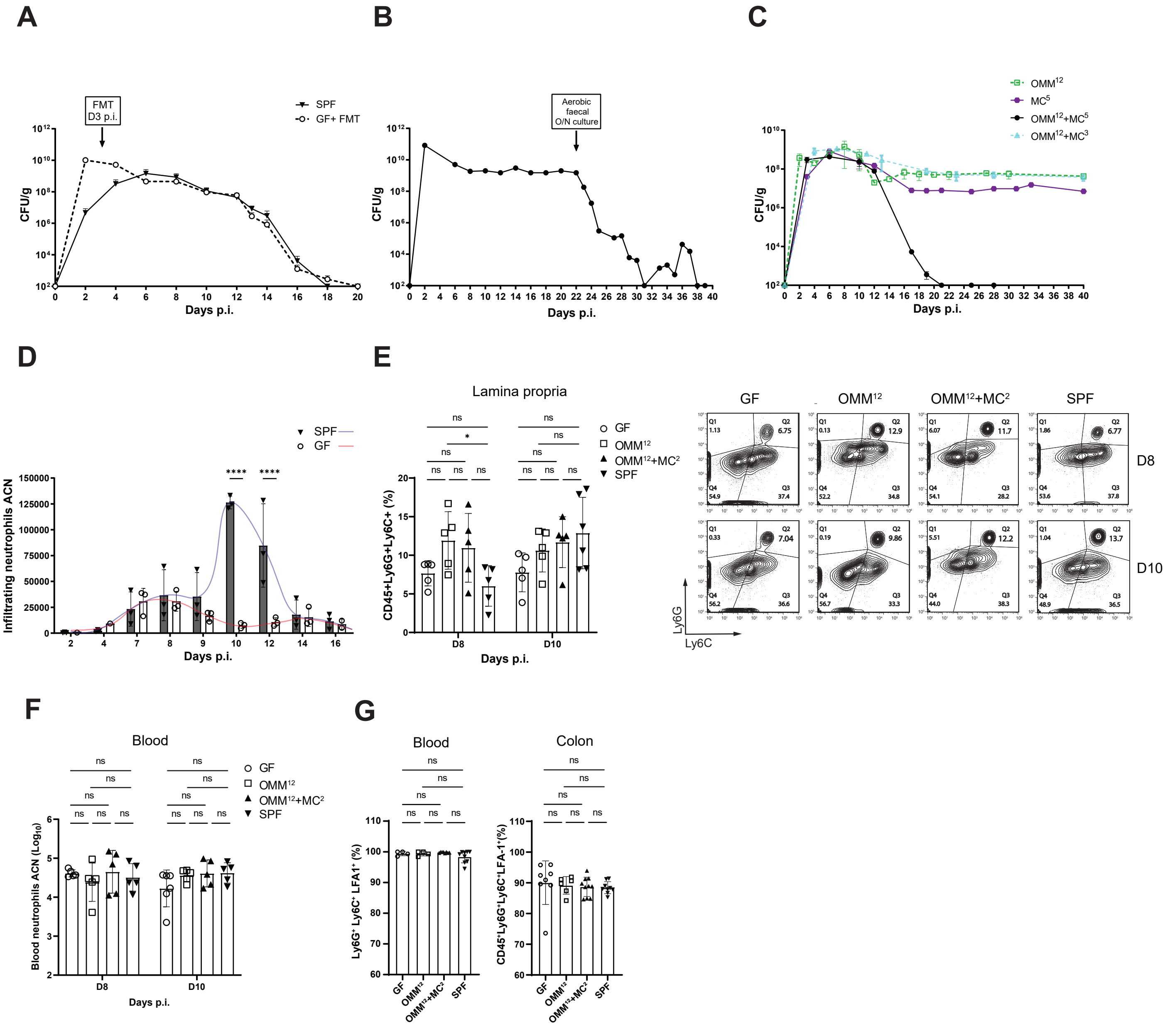

Supplemental 2

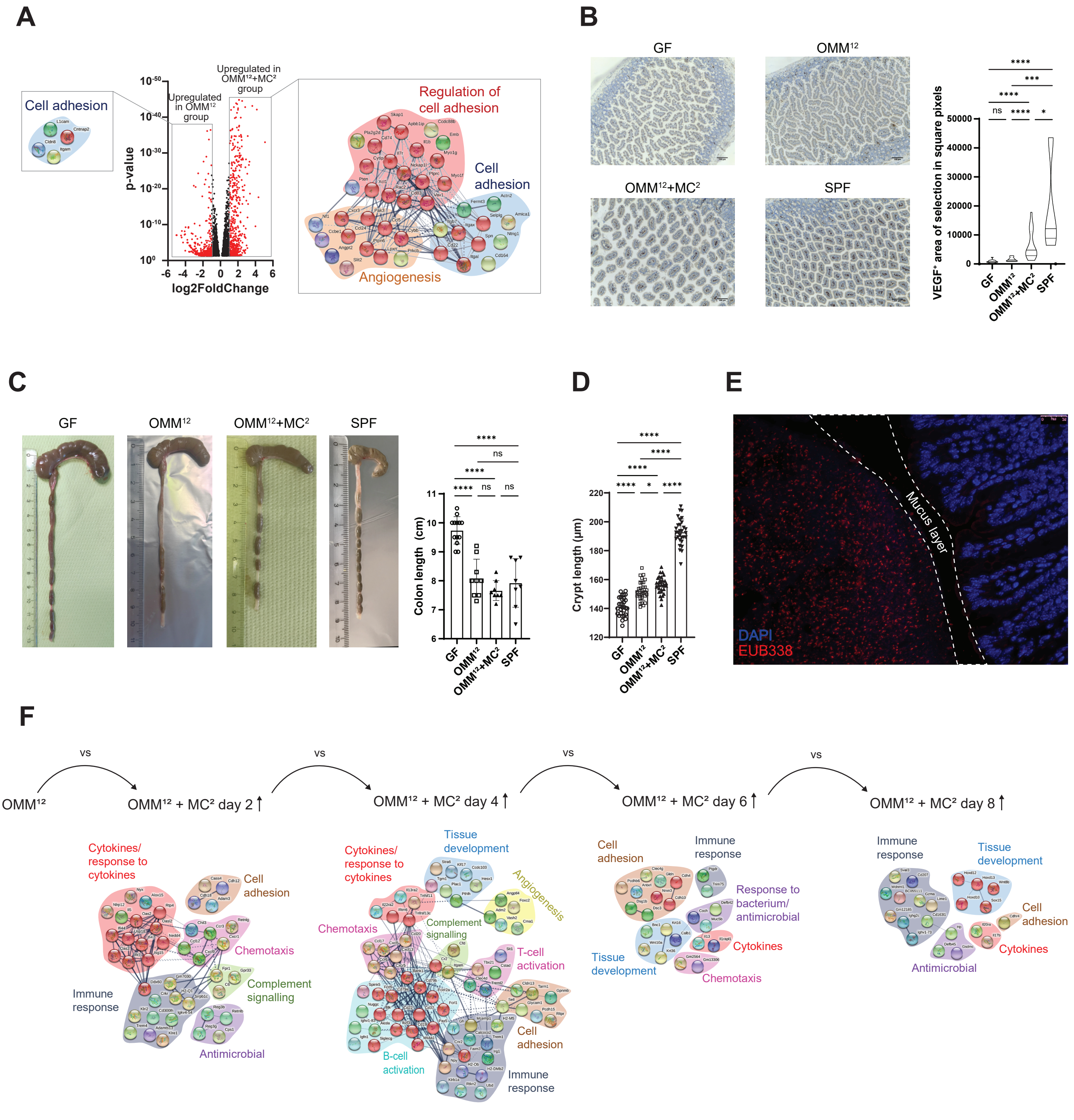

Supplemental 3

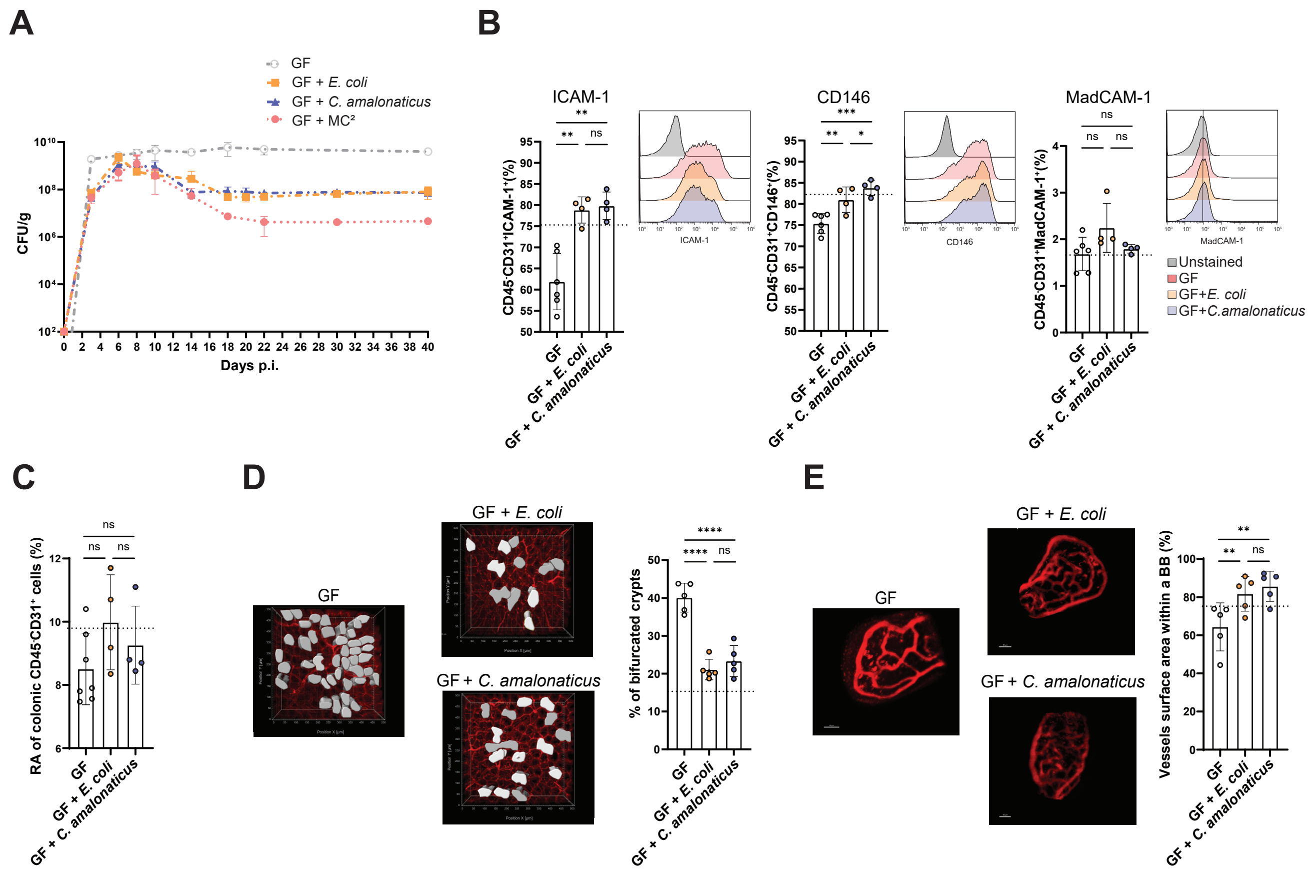

Supplemental 4

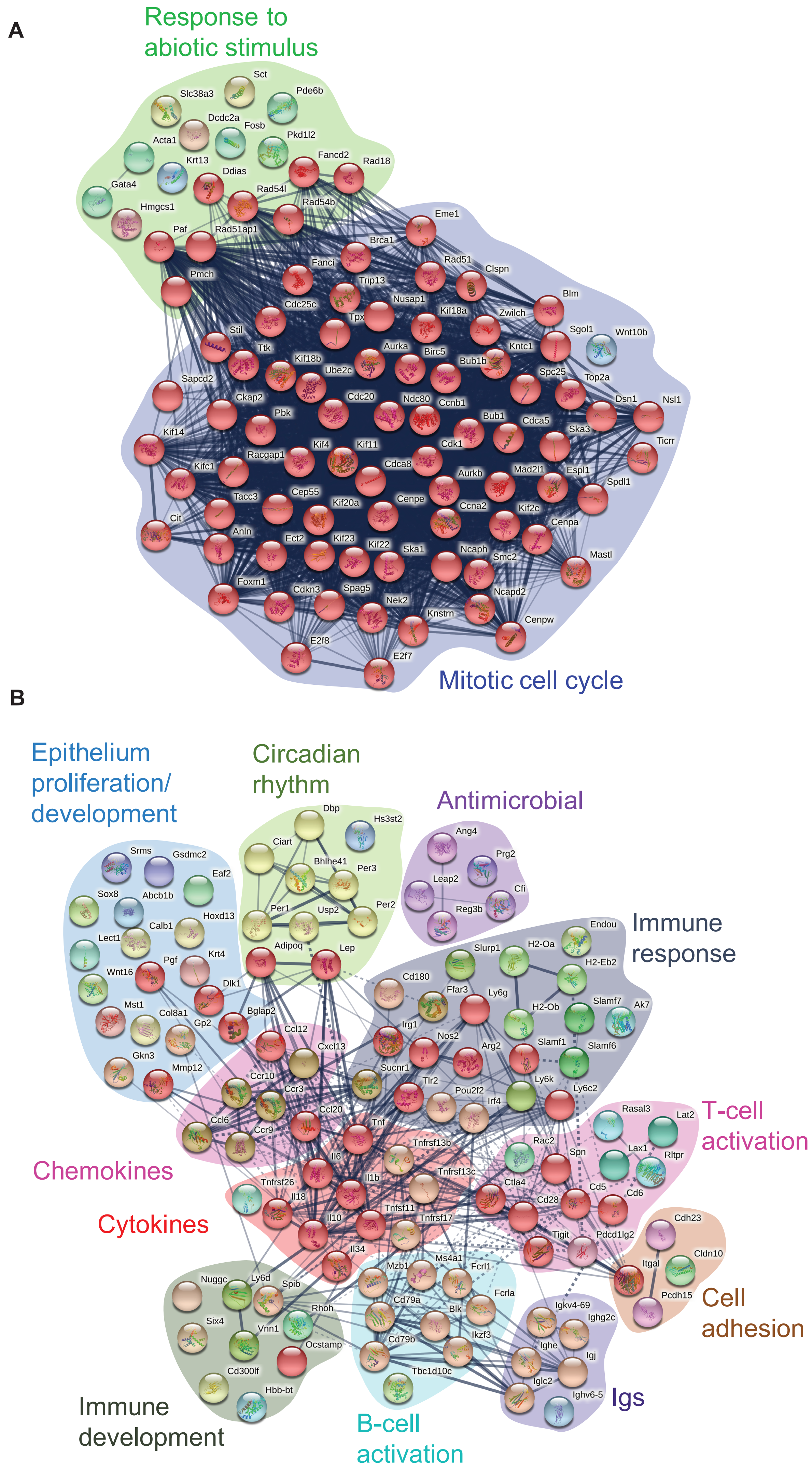

Supplement: Supplementary file 2 — Additional file 1: Figure S1. Microbiota determines neutrophil mediated elimination of C. rodentium. (A) Fecal CFU in C. rodentium infected SPF and GF mice, after fecal microbiota transplant (FMT) from SPF mice. (B) C. rodentium-carriers were gavaged on day 22 p.i with an overnight (O/N) aerobic culture of feces from SPF animals, CFUs were determined. (C) Fecal CFUs of C. rodentium in indicated mice. (D) Kinetic of colonic neutrophils absolute cell numbers (ACN) during the course of C. rodentium infection. (E) Neutrophil frequencies in the colonic lamina propria of indicated mice at day 8 and10 p.i. (F) ACN of neutrophils in the blood of indicated mice at day 8 and 10 p.i. (G) Frequencies of LFA-1+ neutrophils in the blood and colon of C. rodentium infected mice. For C. rodentium infection: animals were orally infected with (109 CFU/ml) C. rodentium. Data shown are means ±SD and representative of at least two independent experiments, each symbol represents the individual value for one mouse (G-I). One-way ANOVA was used: ****p < 0.0001; ** p ≤ 0.01; * p ≤ 0.05; ns, not significant. Figure S2. Commensal triggered gene expression and macroscopic changes of the colon. (A) Volcano plot showing fold-change of gene expression in colonic endothelial cells of OMM12 and OMM12 +MC2 (log2-fold change≥1; p-adj < 0.05). STRING analysis for significantly upregulated transcripts in colonic endothelial cells of OMM12 (left) and OMM12 + MC2 (right) mice. (B) Representative immunostaining and quantification of VEGFa expression (brown staining) in the ileum of indicated mice. (C) Macroscopic alterations and colon length in response to the microbiota. (D) Quantification of colonic crypt length in indicated mice, each symbol represents one crypt (n=3). (E) OMM12+MC2 bacteria were visualized by FISH in medial colon (red, bacteria; blue, DAPI). (F) STRING analysis of differentially expressed genes (log2-fold change ≥1; p-adj < 0.05) in complete colon of OMM12 after colonisation with MC2 ( [file 40168_2022_1353_MOESM1_ESM.pdf]
