# Supplementary material for: Selected commensals educate the intestinal vascular and immune system for immunocompetence
Source: Microbiome. 2022 Sep 28;10:158. doi: 10.1186/s40168-022-01353-5 (PMC9520927; doi:10.1186/s40168-022-01353-5)
Supplement: Supplementary file 4 — Additional file 3. [file 40168_2022_1353_MOESM3_ESM.docx]

**Additional information for bacterial sequencing:**

Bacterial genomic DNA was extracted using the PureLink Genomic DNA Mini Kit (Invitrogen) as recommended by the manufacturer.

Long read whole genome sequencing library preparation was performed using Oxford Nanopore Technologies ligation based sequencing protocol (Native barcoding genomic DNA with EXP-NBD104 and SQK-LSK109) as advised by the manufacturer. For long read sequencing MinION R9.4.1 Flow Cell and Mk1c device have been used.

Short read whole genome sequencing library preparation was performed using Nextera XT protocol with DNA UD Indexes Set D and 1% PhiX. For 150 bp paired-end sequencing Illumina MiSeq device and Micro Flow Cell v2 have been used.

High accuracy basecalling and demultiplexing of long read data was performed with ONT Guppy software v6.1.2+e0556ff93 (Model: dna_r9.4.1_450bps_hac, <https://community.nanoporetech.com/downloads>).

Long read assembly was performed with Flye v.2.9-b1768 [Kolmogorov, M., Yuan, J., Lin, Y. *et al.* Assembly of long, error-prone reads using repeat graphs. *Nat Biotechnol* **37,** 540–546 (2019). https://doi.org/10.1038/s41587-019-0072-8 ].

Medaka v1.5.0 (<https://github.com/nanoporetech/medaka>) was applied for polishing with long reads and for further polishing steps of the draft assembly the generated short read data was used in combination with bwa-mem2 v2.2.1 & Polypolish v0.5.0 as well as POLCA (from MaSuRCA v4.0.5) [Vasimuddin Md, Sanchit Misra, Heng Li, Srinivas Aluru. Efficient Architecture-Aware Acceleration of BWA-MEM for Multicore Systems. *IEEE Parallel and Distributed Processing Symposium (IPDPS), 2019.* , Wick RR, Holt KE (2022) Polypolish: Short-read polishing of long-read bacterial genome assemblies. PLoS Comput Biol 18(1): e1009802. https://doi.org/10.1371/journal.pcbi.1009802 , Zimin AV, Salzberg SL. The genome polishing tool POLCA makes fast and accurate corrections in genome assemblies. PLoS computational biology. 2020 Jun 26;16(6):e1007981. , Zimin AV, Marçais G, Puiu D, Roberts M, Salzberg SL, Yorke JA. The MaSuRCA genome assembler. Bioinformatics. 2013 Nov 1;29(21):2669-77. ].

Rearrangement of the assembly was done via Circlator v1.5.5 [Hunt, M., Silva, N.D., Otto, T.D. *et al.* Circlator: automated circularization of genome assemblies using long sequencing reads. *Genome Biol* **16,** 294 (2015). <https://doi.org/10.1186/s13059-015-0849-0>].

NCBI PGAP v6.1 (online) was used for general annotation during genome submission process [Tatusova, T., DiCuccio, M., Badretdin, A., Chetvernin, V., Nawrocki, E. P., Zaslavsky, L., Lomsadze, A., Pruitt, K. D., Borodovsky, M., & Ostell, J. (2016). NCBI prokaryotic genome annotation pipeline. *Nucleic acids research*, *44*(14), 6614–6624. https://doi.org/10.1093/nar/gkw569 ].

Furthermore, specific analysis of virulence factor genes has been done with blastp v2.12.0+ and virulence factor database (VFDB core/set A update 27^th^ of May 2022, identity >=50%, alignment-length/query-length coverage >= 50%, alignment-length/subject-length coverage >= 50%, best hit per locus based on bitscore) [Zheng Zhang, Scott Schwartz, Lukas Wagner, and Webb Miller (2000), "A greedy algorithm for aligning DNA sequences", J Comput Biol 2000; 7(1-2):203-14. , Liu B, Zheng D, Zhou S, Chen L, Yang J. VFDB 2022: a general classification scheme for bacterial virulence factors. *Nucleic Acids Res*. 2022;50(D1):D912-D917. doi:10.1093/nar/gkab1107].

The analysis and transformation of raw data output has been done with R v4.2.0 [R Core Team (2022). R: A language and environment for statistical computing. R Foundation for Statistical Computing, Vienna, Austria. https://www.R-project.org/].

For the detection of antibiotic resistance genes, RGI v5.2.1 and CARD database v3.2.2 were applied [Alcock BP, Raphenya AR, Lau TTY, et al. CARD 2020: antibiotic resistome surveillance with the comprehensive antibiotic resistance database. *Nucleic Acids Res*. 2020;48(D1):D517-D525. doi:10.1093/nar/gkz935].

Finally, genome based bacterial species identification was done via Type Strain Genome Server and KmerFinder v3.2 [vgl. **TYGS_Refs** , vgl. **CGE_KmerFinder_Refs** ].

**Reference lists**

**TYGS_Refs**:

[1] Meier-Kolthoff JP, Göker M. TYGS is an automated high-throughput platform for state-of-the-art genome-based taxonomy. Nat. Commun. 2019;10: 2182. DOI: 10.1038/s41467-019-10210-3

[2] Meier-Kolthoff JP, Sardà Carbasse J, Peinado-Olarte RL, Göker M. TYGS and LPSN: a database tandem for fast and reliable genome-based classification and nomenclature of prokaryotes. Nucleic Acid Res. 2022;50: D801–D807. DOI: 10.1093/nar/gkab902

**CGE_KmerFinder_Refs**:

1. Hasman H, Saputra D, Sicheritz-Ponten T, Lund O, Svendsen CA, Frimodt-Møller N, Aarestrup FM. (2014). Rapid whole-genome sequencing for detection and characterization of microorganisms directly from clinical samples.

Journal of Clinical Microbiology, Jan;52(1):139-46.

2. Larsen MV, Cosentino S, Lukjancenko O, Saputra D, Rasmussen S, Hasman H, Sicheritz-Ponten T, Aarestrup FM, Ussery DW, Lund O. (2014). Benchmarking of methods for genomic taxonomy.

Journal of Clinical Microbiology, May;52(5):1529-39.

3. Clausen PTLC, Aarestrup FM, Lund O. (2018). Rapid and precise alignment of raw reads against redundant databases with KMA.

BMC Bioinformatics, 19(1):307.

**Results:**

The genome of *Escherichia coli* RSHH22 consists of a circular chromosome of 5,094,340 bp and two extrachromosomal circular sequences designated as plasmid pEC_RSHH22_1 (161,535 bp) and plasmid pEC_RSHH22_2 (4,538 bp). The genome harbours 5,169 genes in total. We identified eigth 5S and seven 16S and 23S rRNA genes. 89 tRNA genes were detected.

The genome of *Citrobacter amalonaticus* RSHH22 consists of a circular chromosome of 4,877,251 bp and one extrachromosomal circular sequence designated as plasmid pCA_RSHH22_1 (110,958 bp). The genome harbours 4,808 genes in total. For *Citrobacter amalonaticus* RSHH22 we identified eigth 5S and seven 16S and 23S rRNA genes. 87 tRNA genes were detected.

Annotation details regarding potential virulence factor genes and antibiotic resistance genes can be found in additional file Supplemental Table 1.

**Data availability:**

Genome sequence data for *Escherichia coli* RSHH22 and *Citrobacter amalonaticus* RSHH22 have been submitted to the NCBI under the accession numbers CP096902-CP096904 and CP096905-CP096906.
